# Profiling of Volatile Organic Compounds from Four Plant Growth-Promoting Rhizobacteria by SPME–GC–MS: A Metabolomics Study

**DOI:** 10.3390/metabo12080763

**Published:** 2022-08-19

**Authors:** Msizi I. Mhlongo, Lizelle A. Piater, Ian A. Dubery

**Affiliations:** Research Centre for Plant Metabolomics, Department of Biochemistry, University of Johannesburg, Auckland Park, P.O. Box 524, Johannesburg 2006, South Africa

**Keywords:** metabolomics profiling, multivariate data analysis (MVDA), plant growth promoting rhizobacteria (PGPR), solid-phase micro-extraction gas chromatography mass spectrometry (SPME–GC–MS), volatile organic compounds (VOCs)

## Abstract

The rhizosphere microbiome is a major determinant of plant health. Plant-beneficial or plant growth-promoting rhizobacteria (PGPR) influence plant growth, plant development and adaptive responses, such as induced resistance/priming. These new eco-friendly choices have highlighted volatile organic compounds (biogenic VOCs) as a potentially inexpensive, effective and efficient substitute for the use of agrochemicals. Secreted bacterial VOCs are low molecular weight lipophilic compounds with a low boiling point and high vapor pressures. As such, they can act as short- or long-distance signals in the rhizosphere, affecting competing microorganisms and impacting plant health. In this study, secreted VOCs from four PGPR strains (*Pseudomonas koreensis* (N19)*, Ps. fluorescens* (N04)*, Lysinibacillus sphaericus* (T19) and *Paenibacillus alvei* (T22)) were profiled by solid-phase micro-extraction gas chromatography mass spectrometry (SPME–GC–MS) combined with a multivariate data analysis. Metabolomic profiling with chemometric analyses revealed novel data on the composition of the secreted VOC blends of the four PGPR strains. Of the 121 annotated metabolites, most are known as bioactives which are able to affect metabolism in plant hosts. These VOCs belong to the following classes: alcohols, aldehydes, ketones, alkanes, alkenes, acids, amines, salicylic acid derivatives, pyrazines, furans, sulfides and terpenoids. The results further demonstrated the presence of species-specific and strain-specific VOCs, characterized by either the absence or presence of specific VOCs in the different strains. These molecules could be further investigated as biomarkers for the classification of an organism as a PGPR and selection for agricultural use.

## 1. Introduction

Plant growth-promoting rhizobacteria (PGPR), part of the plant microbiome in the rhizosphere, is a community that is largely influenced by plant roots and soil type [1,2,3]. The roots secrete molecules as part of the root exudates, and these inadvertently influence the microbial consortium in the adjacent rhizosphere [4,5,6]. In agroecosystems, PGPR has been shown to influence plant growth by improving soil nutrients, inhibiting deleterious organisms and enhancing plant health [1,3]. Numerous strains have been reported to interact with plants (below and above ground), resulting in either antagonistic or mutualistic symbiosis [7,8,9,10]. To help plants defend against the plethora of pathogens, PGPR can elicit a form of resistance known as induced systemic resistance (ISR) [11,12]. Symbiotic interactions have been thought to involve physical contact between plants and PGPR. Nonetheless, recent studies have reported that microbial signals (i.e., volatile organic compounds or VOCs) play an integral role in multi-trophic interactions with plants. These include functions in plant growth modulation and health, as well as enhancement of the availability of soil nutrients and interactions with other microbial communities within the rhizosphere [13,14,15].

PGPR coexist in dynamic communities and secrete VOCs that allow these organisms to survive in their environments [10]. As low molecular weight compounds and easily vaporized molecules, they can diffuse through complex matrixes, such as cellular membranes, water, soil and air [10,16,17]. VOCs are thus suitably equipped as info-chemicals produced by soil microbes that mediate both short- and long-distance signaling, as well as inter- and intra-organismic communication below and above ground [9,10,15,16,17,18,19]. Plant growth enhancement and physiological processes, such as ISR and abiotic stress tolerance after VOC exposure (summarized in Figure S1), are dependent on systemic changes modulated by alterations in the levels of phytohormone, such as ethylene (ET), auxin and jasmonic acid (JA). Still, other phytohormones and concomitant cross-talk cannot be excluded [19,20,21]. Studies suggest that the inoculation of plant roots with PGPR or exposure to VOCs involves several signaling pathways that lead to the stimulation of photosynthesis and plant growth, cell wall modification, phytohormone regulation and stress responses [22,23]. In addition, PGPR VOCs can influence rhizosphere communities in either a positive or negative manner through bacterial–bacterial and bacterial–fungi interactions [10]. For example, these can either recruit other beneficial microbes, inhibit pathogenic microbes or attract natural enemies for feeding on soil-borne herbivores [24,25].

Multiple signals are responsible for the successful colonization of plant roots by PGPR in a complex process controlled by molecules from the two organisms involved. In this study, four bacterial strains with PGPR activity (*Pseudomonas koreensis* (N19), *Ps. fluorescens* (N04)*, Lysinibacillus sphaericus* (T19) and *Paenibacillus alvei* (T22)) were investigated. Previously, these PGPRs have been reported to successfully colonize maize, wheat and tomato roots, and to enhance the growth of these plants [26,27]. Some of the elucidated mechanisms by which these bacterial isolates stimulate plant growth include the production of indole-acetic acid and related hormones, phosphate solubilisation and the production of siderophores [26]. Lastly, *Pa. alvei* (T22) was also found to be effective as a biocontrol agent against soil-borne diseases in sorghum and wheat [28,29].

A previous study investigating the four PGPR strains revealed strain-specific defence-related metabolic reprogramming in tomato plants [30]. The obtained results suggested that the differential modulation of the identified metabolites (apparent from altered metabolic profiles in response to the four PGPR strains) was dependent on bacteria-derived stimuli and that the plants adjusted their adaptive responses based on the perceived signal(s). As such, the aim of this investigation was to fill the gap in our understanding of how these strains promote growth and induce resistance in crop plants. This was achieved by profiling the secreted VOCs and comparing them to PGPR strains that were reported to have these effects on plants.

Due to their volatile nature, VOCs are appropriately analyzed by gas chromatography for separation coupled to mass spectrometry for detection and identification (GC–MS). The non-destructive headspace (HS) sampling strategy is a sensitive extraction method for detecting and analyzing natural volatile compounds, and various methods (either dynamic or static) have been developed. In this regard, procedures such as stir bar sorptive extraction, in-tube extraction and solid phase micro-extraction (SPME) fibers have been applied for HS analysis [31,32,33]. As a solvent-free extraction method, SPME integrates the sample preparation stages (sampling, extraction, concentration and sample introduction) into a single step [34,35]. This significantly decreases preparation time and provides samples ready for analysis. Different needle-coating chemicals such as polydimethylsiloxane, carboxen, divinylbenzene, polyacrylonitrile and benzenesulfonic acid have been developed as sorptive phases for different analyte classes [35,36]. Coatings with different thickness and polarities also broaden fiber selection [36]. Notwithstanding all the advantages offered by SPME, the chemical base coating of the stationary phase can limit sensitivity or favor certain classes of analytes based on their size and/or polarity [37,38].

## 2. Materials and Methods

### 2.1. Bacterial Growth and Experimental Design

The bacterial strains (*Pseudomonas koreensis* (N19)*, Ps. fluorescens* (N04), *Lysinibacillus sphaericus* (T19) and *Paenibacillus alvei* (T22)), were obtained from the PGPR culture collection of Professor N. Labuschagne, University of Pretoria, South Africa [26,27]. The strains were cultivated individually in Luria-Bertani (LB) medium at 28 °C and stocks were maintained in LB medium containing 15% (*v/v*) glycerol at −80 °C. Gas chromatography coupled to a time-of-flight mass spectrometer (GC–TOF–MS) was used to profile volatiles secreted by these organisms in a non-targeted manner. The experiments were designed in such a way to be correct for non-targeted metabolomic analysis in conjunction with multivariate data analysis (i.e., three biological replicates, with each sample analyzed in triplicate (n = 9)).

### 2.2. Volatile Collection and Headspace Sampling through SPME

We inoculated 50 mL of LB broth in 250 mL Erlenmeyer flasks with a 50 µL suspension of each PGPR strain. These samples were cultured overnight at 28 °C on an orbital shaker (Labotec, Midrand, South Africa). Subsequently, 10 mL of the overnight cultures were transferred into 20 mL sample vials (22 cm × 7.5 cm) and were further incubated under the same conditions until saturation (OD_600_ > 2) was reached. LB broth without any inoculation was used as the control. Bacterial VOCs were extracted from the headspace of the 20 mL vials and were concentrated via SPME before desorption in the GC injection port. All samples were sequentially incubated at 50 °C in an incubator set at 300 rpm for 30 min before VOC extraction and were kept at this temperature throughout sampling in order to assist the release of the VOCs from the aqueous media. The SPME fibers used were fused silica fibers with a polydimethylsiloxane/divinylbenzene (PDMS/DVB) coating (Supelco, Munich; Germany). These were used to pierce the polytetrafluoroethylene (PTFE) septa and expose the fibers to the headspace of the vial for 10 min. Immediately after VOC extraction, the SPME fibers were inserted into the GC injection port for 1 min and were exposed to 200 °C for the desorption of the adsorbed VOCs. For reconditioning, the fibers were heated at 250 °C for 10 min.

### 2.3. Gas Chromatography—Time-of-Flight—Mass Spectrometric Analysis

For GC–MS analyses, a Pegasus BT GC–TOF–MS (LECO Corporation, St. Joseph, MI, USA) fitted with a Rx-5MS column (30 m, 0.25 mm ID, 0.25 µm film thickness df) (Restek, Bellefonte, PA, USA) instrument was used. SPME fibers were desorbed at 200 °C for 1 min in the injection port. Helium was used as a carrier gas, with a total flow rate of 14 mL/min and column flow rate of 1 mL/min. GC–MS runs were 25 min, with the fiber remaining in the injection port for 10 min after each run. The injection port was operated in a ‘splitless’ mode, with a constant He flow of 1.0 mL/min. The initial oven temperature was 50 °C and was held at this temperature for 1 min before it was ramped up to 280 °C (with an increase of 15 °C/min) and was held at this temperature for 3 min. The high-resolution (HR) time-of-flight mass spectrometer (TOF–MS) parameters were as described below. The data acquisition rate was 20 scans/s and the extraction frequency was 30 kHz. The ion source temperature was 200 °C and the interface temperature was 200 °C. The detector voltage was related to the tuning file or 70 eV, and the mass-to-charge range (*m/z*) was from 35 to 500.

### 2.4. Multivariate Data Analysis (MVDA)

The acquired high-resolution GC–TOF–MS datasets were converted to NetCDF files using LECO ChromaTOF-HRT software, version 5.0. These were exported to the XCMS online statistical package (https://xcmsonline.scripps.edu, accessed on 10 November 2019), an automated, web-based metabolomics data processing software for peak picking and peak alignment. The parameters for the method were selected for GC–TOF–MS specificities and were as follows: (i) feature detection was set as the centWave method, minimum peak width = 0.6, maximum peak width = 1.0; (ii) Rt correction was set as the Obiwarp method, Profstep = 1, alignment was set as *m/z* width = 0.015, minfraction = 0.5 and bw = 5. The resulting peak list showed 7550 variables (in a combined list) with corrected peak retention times (Rts, min), mass-to-charge ratios (*m/z*) and integrated peak areas.

The resultant data matrixes obtained from the XCMS statistical package were imported into SIMCA-P software, version 15.0 (Sartorius, Umeå, Sweden) and Pareto-scaling was applied for the multivariate statistical analysis. Firstly, to reduce the dimensionality of the data and to summarize the information contained in the datasets, a principal component analysis (PCA, an unsupervised method for MVDA) was used. Here, the Hotelling’s T-squared (T^2^) test was applied to test for significant differences between the mean vectors (multivariate means) of the multivariate data sets. Secondly, to complement the PCA, a hierarchical cluster analysis (HCA) was used to assess the trends and patterns observed on the PCA [39,40,41].

Orthogonal partial least squares discriminant analysis (OPLS-DA), a supervised MVDA method, was used to extract the maximum amount of information regarding the significant variables from the datasets. This procedure allows for the removal of systematic variation from the experimental data (X variables) that is not correlated to discriminant classes (Y) and Hotelling’s region, represented by the ellipse and defined by the 95% interval of confidence. The quality of the generated models is described by diagnostic tools for metabolomics, which included: (i) cumulative model variation in the matrix X, (ii) the goodness-of-fit parameter [R^2^X(cum)], (iii) the model variance proportion [Y^2^X(cum)] and (iv) total variation of the matrix X predicted by an extracted component [Q^2^(cum)] [42].

In addition, the OPLS-DA models were statistically validated using a cross-validated analysis of variance (CV-ANOVA), with a *p* ≤ 0.05 indicating a good model [43], using the SIMCA inbuilt 7-fold (default) CV method and a permutation test (50 permutations). OPLS-DA S-plots were constructed for subsequent interpretation. Based on the S-plots, significant *m/z* ions with a correlation of [(*p*(*corr*)] ≥ |0.5| and a covariance of (*p*1) ≥ |0.05| were selected. These ions were further validated using ‘variable importance in projection’ (VIP) scores of >1 before selection for annotation. Statistically significant features were annotated (tentatively identified) to level 2, as defined by the metabolomics standards initiative [44], based on their mass spectral information using the National Institute of Standards and Technology (NIST) Mainlib [45], and Fiehn metabolomics and Mass Spectrum libraries [46], selecting compounds with a similarity of 75% and above.

## 3. Results

The composition of non-selective extracted metabolomes are complex due to their chemo-diversity. This multidimensionality presents holdups in metabolomics studies. However, recent high-resolution (HR) technological advancements, such as the GC–HR–time-of-flight–(TOF)–MS, have enabled researchers to concurrently detect multiple analytes with high sensitivity, thus providing more detailed information about the metabolic profile of the sample. Some of the advantages of the GC–TOF–MS include: (i) the deconvolution power of the software, allowing the detection and resolution of overlapping peaks within seconds and (ii) high-speed data acquisition without distorting the peak height and constant MS spectral scans across a peak, regardless of peak intensity, to assist with annotation. Visual inspection of the generated MS chromatograms showed differential profiles of the analyzed samples with characterized unique peak populations (absent, present and/or differing in peak intensity), thus info-graphically reflecting variances among the sampled VOCs from the four PGPR strains (Figure S2). Data pre-processing [47] and comparative chemometrics [48] were used to extract the underlying features that contribute to the observed differences.

**Figure 1:**
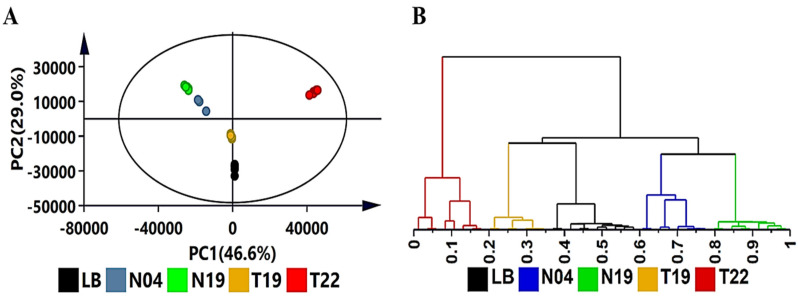
Exploratory data analysis of volatile organic compounds produced by the four PGPR strains (N19, N04, T19, T22) with unsupervised chemometric methods. LB = control comprising of uninoculated Luria-Bertani medium. (**A**): A PCA scores scatter plot of all the samples colored according to PGPR strain. The ellipse represents the Hotelling’s T^2^ distribution with 95% confidence. The PCA model presented here was a 5-component model, with R^2^X of 93.6% and Q^2^(cum) of 91.3%. (**B**): The HCA dendrogram corresponding to the PC analysis. Unsupervised statistical analysis is used to generate the subgrouping of samples based on similar observations in (**A**), whereas the HCA dendrogram shows the hierarchical relationship between samples (**B**).

Following data pre-processing, the three-dimensional (Rt, *m/z* ratio and peak area) output that was created was exported to software (SIMCA Data Analytics software) for further multivariate statistical analysis. PCA models were computed in order to compare the volatile profiles of the four strains investigated. PCA, as an unsupervised modeling tool for data exploration, reduced the dimensionality of the data and allowed for inspection of the global distribution patterns (clustering of sample groups, trends and outliers). The computed PCA (Figure 1A) shows sample grouping of the various strains and the characterization of the volatile profiles. It is important to note that the two *Pseudomonas* strains (*Ps. koreensis* (N19) and *Ps. fluorescens* (N04)) are clustered next to each other, which could indicate that these have similar profiles, whereas *Pa. alvei* (T19) was found to cluster nearer to the uninoculated LB media. *L. sphaericus* (T22) was found to cluster on its own further away from the rest, indicating that the volatile profile of this strain is significantly different from that of the other strains. The PCA-extracted trends and patterns were further examined by HCA for comparative exploration. The HCA models were computed using the linkage method of Ward, considering ‘between’ and ‘within’ cluster distances. The trees were sorted based on the size of the clusters [39,40,41]. The computed HCA (Figure 1B) shows two major clusters of *L. sphaericus* (T22) vs. the other samples, where the latter are further divided into two subgroups: LB (medium control) and *Pa. alvei* (T19), and the two *Pseudomonas* strains (N04 and N19).

**Figure 2:**
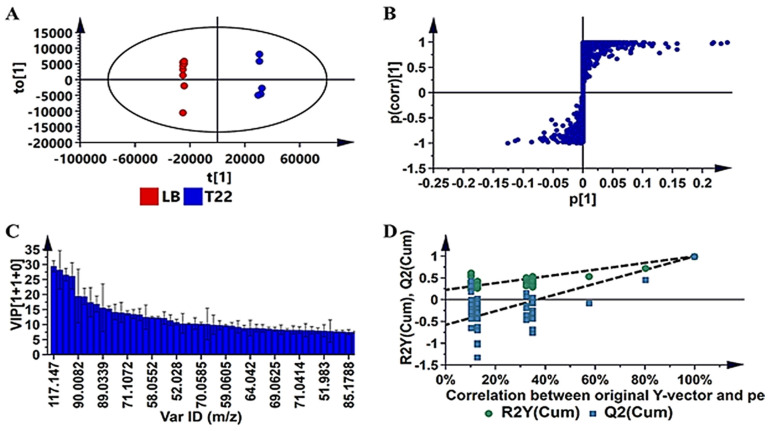
OPLS-DA modeling and variable/feature selection of *Lysinibacillus sphaericus* (T22) data acquired on GC–TOF–MS. (**A**): A typical OPLS-DA score separating uninoculated media (LB) vs. inoculated media (T22) (1 + 1 + 0 components, R^2^X = 0.895, Q^2^ = 0.999, CV-ANOVA *p*-value = 1.3 × 10^−13^). (**B**): An OPLS-DA loadings S-plot for the same model in (**A**); only variables with a correlation [*p*(*corr*)] of ≥|0.5| and a covariance of (*p*1) ≥ |0.05| were chosen as discriminating variables and were identified using the *m/z* to generate elemental composition. (**C**): A variable importance for the projection (VIP) plot for the same model, pointing mathematically to the importance of each variable in contributing to group separation in the OPLS-DA model. (**D**): The response permutation test plot (n = 50) for the same OPLS-DA model: R^2^ (0.0, 0.207) and Q^2^ (0.0−0.739) values of the permuted models are represented on the left-hand side of the plot, corresponding to *y*-axis intercepts. Similar figures for VOCs secreted by N04, N19 and T19 are presented in Figures S3–S5.

Although the models generated from unsupervised PCA and HCA revealed the structures within/between the datasets, the modeling lacks predictive power. Hence, this modeling was followed by an alternative method, namely orthogonal projection to latent discriminant analysis (OPLS-DA). OPLS-DA models were calculated using two predefined conditions (LB vs. each bacterial strain) in order to extract differences in the samples under investigation so as to assist with the identification of features that were responsible for the observed dissimilarities [48,49]. As an example, the OPLS-DA score plot for *L. sphaericus* (T22) (Figure 2A) shows distinct separation of the inoculated sample from the uninoculated sample. The corresponding S-plot (Figure 2B) was used to identify features that positively correlated to the various inoculations.

To prevent bias in selecting significant features, variable importance in projection (VIP) plots were constructed to rank the importance of individual variables to the models [48,49]. As such, VIP plots (Figure 2C) were used to validate the variable selection based on the S-plots, and only variables with a VIP score of >1 were selected for annotation—these are summarized in Table S1. Furthermore, to validate the predictive capability of the computed OPLS-DA models, a response permutation test (with n = 50) (Figure 2D) was used. In this statistical test, the R^2^ and Q^2^ values of the true model are compared with that of the permutated model. The test was carried out by random assignment of the dataset rows to two different groups, after which the OPLS-DA models were fitted to each permutated class variable. The R^2^ and Q^2^ values were then computed for the permutated models and compared to the values of the true models. The model validity was supported by having all Q^2^ values of the permuted dataset (to the left) lower than the Q^2^ value on the actual data set (to the right). Furthermore, the regression line (the line joining the point of observed Q^2^ to the centroid of a cluster of permuted Q^2^ values) has a negative intercept value on the *Y*-axis [50,51]. The *y*-axis displays R^2^ and Q^2^, whereas the *x*-axis shows the correlation coefficient of the permuted and observed data. The two points on the right represent the observed R^2^ and Q^2^. The green and blue dots represent R^2^ and Q^2^ values, respectively. The dashed lines indicate the corresponding fitted regression lines for the observed and permutated R^2^ and Q^2^.

**Figure 3:**
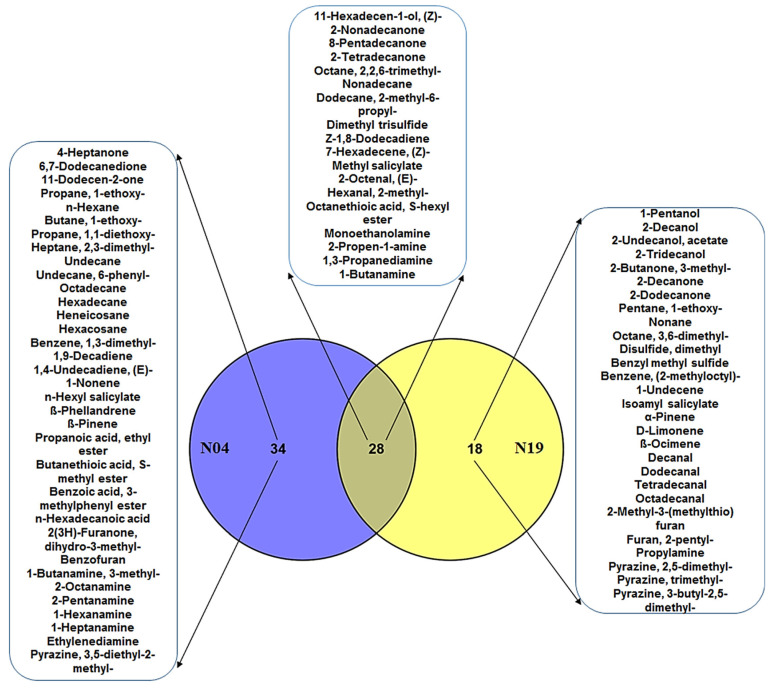
Venn diagram comparing the volatile organic compounds of the two *Pseudomonas* strains. The diagram shows shared and distinct volatile organic compounds from *Pseudomonas koreensis* (N19) and *Pseudomonas fluorescens* (N04), as indicated by the numbers in the intersections and ellipses, respectively.

**Figure 4:**
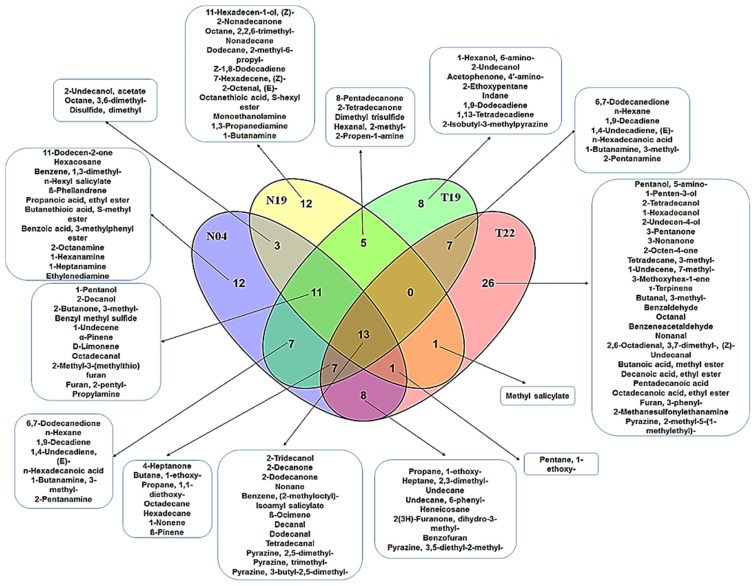
Venn diagram of volatile organic compounds produced by *Pseudomonas* strains (*Pseudomonas koreensis* (N19) and *Pseudomonas fluorescens* (N04)), vs. *Paenibacillus alvei* (T22) and *Lysinibacillus sphaericus* (T19). The diagram shows overlapping and distinct VOCs, indicated by the numbers in the intersections and ellipses, respectively.

PGPR produce a large spectrum of VOCs, contributing to their ability to interact with and influence neighboring organisms or plants. The ‘volatilome’ of a specific strain consists of wide and diverse VOCs with regards to their chemical and physical properties. The composition and relative abundance of the VOC blend is highly dependent on the specific strain, but also on the growth phase and growth conditions [52,53]. Moreover, the emission of specific VOCs can be induced or influenced by interacting organisms present in the bacterial environment. In general, closely related species emit a similar blend of VOCs (with some species-specific metabolites) compared to distantly related species [52]. Using SPME–GC–TOF–MS to profile the VOCs produced by the four PGPR strains (*Ps. fluorescens* (N04), *Ps. koreensis* (N19), *Pa. alvei* (T19) and *L. sphaericus* (T22)), a total of 121 VOCs (Table S1) were identified. The annotated VOCs generally belong to the following classes: aldehydes, alcohols, ketones, acids, alkanes, alkenes, amines, derivatives of salicylic acid, pyrazines, furans, sulfides and terpenoids (Table S1), and most have been previously reported. The two Pseudomonads had 28 VOCs in common (Figure 3), whereas the four strains were found to share only a small number of overlapping molecular patterns among a large pool of different VOCs (Figure 4). These included 2-tridecanol, 2-decanone, 2-dodecanone, nonane, decanal, dodecanal, tetradecanal, isoamyl salicylate, benzene (2-methyloctyl), pyrazine (2,5-dimethyl), pyrazine (trimethyl), pyrazine (3-butyl-2,5-dimethyl) and ß-ocimene. The observed variation in VOC profiles could suggest that these strains promote plant growth and inhibit pathogen growth in different ways.

## 4. Discussion

### 4.1. Comparison and Evaluation of Volatile Organic Compounds Secreted by the Two PGPR Pseudomonas Strains

*Pseudomonas* spp. are one of the most dominating groups in the rhizosphere for which both the promotion of plant growth and antagonistic properties towards pathogens have been documented. Interestingly, studies have shown that bacteria of the same species can exhibit different VOC profiles and have differential perturbation effects on the physiology of plants and microorganisms [15,53]. These have been found to lead to enhanced plant growth and induce resistance and inhibit pathogen growth [26]. In this study, we first compared the profile of two strains of plant protective *Pseudomonas* spp. in order to identify common/unique VOCs (Figure 3). The two strains had 28 VOCs in common, 34 VOCs specific to *Ps. fluorescens* (N04) and 18 VOCs specific to *Ps. koreensis* (N19) (Figure 3). The shared VOCs included methyl salicylate (MeSA), dimethyl trisulfide, nonadecane and 11-hexadecen-1-ol, amongst others. Interestingly, *Ps. fluorescens* (N04) had hexyl salicylate among its unique VOCs, whereas *Ps. koreensis* had isoamyl salicylate as a unique VOC (Figure 3). SA and MeSA are known plant hormones that play different roles in plant growth and development [54]. Accordingly, the ability of these strains to secrete SA derivatives could be one of the mechanisms they employ to establish mutual relationships, enhance plant growth and induce resistance. An interesting molecule worth mentioning is the dimethyl disulfide specific to *Ps. koreensis* (N19), which has been commercialized as a soil fumigant against soil-borne pathogens and nematodes due to its antimicrobial properties and its ability to prevent nematodes from feeding on roots [25].

In addition, the ‘volatilome’ of these strains (common or unique) (Figure 3) consisted of the following classes of compounds: alcohols, ketones, aldehydes, alkanes and sulfides. These classes of VOCs have been reported in various PGPR strains and form part of the complex communication in the rhizosphere that leads to growth stimulation, induced resistance or pathogen growth inhibition. For example, *Pseudomonas* strains that are associated with potato roots produce VOCs with the capacity to greatly inhibit *Phytophthora infestans*, and SPME–GC–MS has shown that these strains secrete similar and unique VOCs that are associated with such inhibition [55]. This report further demonstrated that these strains had varying effects on the mycelial growth of *P. infestans*, thus, indicating that the VOC blend (with regards to its composition and concentration) plays an important role in pathogen inhibition. The VOCs identified in [55] included alcohols, sulfides, alkanes, ketones and aldehydes. Dimethyl disulfide was the only VOC that was found to be secreted in high concentrations. Moreover, when the inhibitory ability of these strains was tested against other plant pathogens, such as *Rhizoctonia solani, Helminthosporium solani, Dickeya dianthicola* and *Fusarium oxysporum,* varying inhibitory effects on the growth of the different phytopathogens were observed [55]. Similarly, VOCs from *Ps. chlororaphis* promoted growth and salt tolerance in Arabidopsis, and 2,3-butanediol from *Ps. chlororaphis* was identified as a VOC that contributed to the induced resistance of tobacco against *Erwinia carotovora*, but not against *Pseudomonas syringae* pv. *tabaci* [21,56].

Analogous to the cited examples, the uniqueness of the VOCs observed in this study (Figure 3) might further explain the different metabolic perturbations caused by these strains in tomato seedlings [57]. In this study, we observed that root inoculation with the four strains induced specific differential metabolic perturbations that were characterized by metabolites from the hydroxycinnamate, benzoate, flavonoid and glycoalkaloid classes. Furthermore, the targeted analysis of aromatic amino acids indicated differential quantitative increases or decreases over two days in response to the four PGPR strains. The observed differences could be a reflection of how effectively the PGPR strains interacted with the tomato roots, as well as the relative activities of specific metabolic pathways that contribute to the annotated VOCs.

### 4.2. Comparison and Evaluation of Volatile Organic Compounds Secreted by the Two Pseudomonas Strains, Paenibacillus alvei (T22) and Lysinibacillus sphaericus (T19)

The VOC profiles of the two *Pseudomonas* strains (*Ps. fluorescens* (N04) and *Ps. koreensis* (N19)) were subsequently analyzed and compared with two PGPR strains from the *Lysinibacillus* (*Ly. Sphaericus* (T19)) and *Paenibacillus* (*Pa. alvei* (T22)) genera in order to evaluate the chemical compositions of the volatile blends and to establish whether these strains secrete similar or unique compounds (Figure 4). An assessment of the annotated VOCs of these different strains showed that they have only 13 metabolites in common (Figure 4), with *Pa. alvei* (T22) having the highest number of unique secreted VOCs under experimental conditions (Figure 4). Moreover, the VOC profiles of the different PGPR strains showed that these species are diverse, as seen by the different combinations of VOCs that were shared by the species and those that were species-specific. The observed differences in the PGPR strains could be an indication that these organisms employ unique mechanisms in order to interact with their neighbors and affect physiological process.

Previous studies have shown that PGPR strains secrete a wide range of VOCs that act as info-chemicals within the bacterial community (among the same species or among different species) or with surrounding organisms [15,58,59]. For example, ref. [59] showed that VOCs produced by four strains of *Bacillus* spp. and *Paenibacillus* spp. had varying antagonistic effects against soil-borne plant pathogens, including *Ascochyta citrullina*, *Alternaria brassicae* and *Al. solani*. Through headspace sampling and GC–MS analyses, an overlapping and unique volatile pattern could be found among the different species.

*Pa. alvei* (T22) has been studied by us for growth promotion and induced resistance against phytopathogens. Treatment of *Sorghum bicolor* seedlings with this strain prior to inoculation with *F. pseudograminearum* [28] and *Colletotrichum sublineolum* [29] significantly lowered the progression of disease by the phytopathogens. In addition, this strain was also effective in inducing a primed state against *Phytophthora capsici* infection in tomato seedlings [60]. As indicated in Figure 4*, Pa. alvei* (T22) secreted the highest number (26) of unique VOCs that possibly contribute to its mechanism of action to induce/enhance the aforementioned resistance.

## 5. Conclusions

Cues originating from mutualistic ecological interactions often contain highly specific information. Recent studies have demonstrated that VOCs emitted by PGPR can be used as a novel agroecological strategy to improve plant growth and yields, as well as to improve resistance to abiotic and biotic stresses. PGPR are members of diverse bacterial genera, and differences in plant protective capabilities can be linked to intrinsic biochemical activities. Advances made in analytical instrumentation have made it possible to resolve analytes present in a complex mixture in a short period of time. Here, SMPE–GC–MS, in combination with chemometrics, were successfully used to profile the VOCs secreted from strains of *Ps. koreensis*, *Ps. fluorescens*, *L. sphaericus* and *Pa. alvei*. Our results indicate that the VOC profiles of rhizobacteria can be quite specific and that even strains of the same species may present unique profiles. These observable differences in the VOC blends can be due to the relative activities of specific metabolic pathways active in these strains. The main groups of VOCs secreted by PGPR include ketones, alkanes, alkenes and alcohols, and these were all identified in the present study. Of the investigated strains, the ‘volatilome’ of *Pa. alvei* (T22) exhibited more unique features in comparison to the others. These annotated molecules could be further investigated as biomarkers for the classification of an organism as a PGPR and their selection for agricultural use.

Currently, efforts to elucidate the mode of action of a specific compound or mixture regulating the plant physiological processes leading to growth, higher yield and inducible protection are underway. Thus, these results support the view that diverse PGPR may affect plant biochemical pathways and processes with different mechanism(s) of action. This might even be applicable if the PGPR belong to the same species, emphasizing the importance of characteristic strain-specific features. In addition, the number of annotated VOCs indicates that there could be more bioactives/volatiles than have been reported thus far. Furthermore, this research highlights the power of high-resolution mass spectrometry for metabolomic application in order to gain deeper insight into the chemical signaling pathways in the rhizosphere and the mechanism(s) of action of plant-protective bacteria. Metabolomic profiling thus offers another tool to functionally dissect the role(s) of VOCs in PGPR priming.

## Data Availability

Data needed to evaluate the conclusions in the paper are present in the paper and the Supplementary Materials. Additional data related to this paper may be requested from the corresponding author.

## Supplementary Materials

The following supporting information can be downloaded at: https://www.mdpi.com/article/10.3390/metabo12080763/s1, Figure S1: Action mechanisms of PGPR-derived VOCs; Figure S2: Representative GC–TOF–MS chromatograms of PGPR volatiles sampled by headspace SPME. Figure S3: OPLS-DA modeling and variable/feature selection of *Pseudomonas fluorescens* (N04) data acquired using GC–TOF–MS. Figure S4: OPLS-DA modeling and variable/feature selection of *Pseudomonas koreensis* (N19) data acquired using GC–TOF–MS. Figure S5: OPLS-DA modeling and variable/feature selection of *Paenibacillus alvei* (T19) data acquired using GC–TOF–MS. Table S1: VOC profiles of PGPR strains: *Pseudomonas fluorescens* (N04), Pseudomonas koreensis (N19), *Paenibacillus alvei* (T19) and *Lysinibacillus sphaericus* (T22).
