# Supplementary material for: Profiling of Volatile Organic Compounds from Four Plant Growth-Promoting Rhizobacteria by SPME–GC–MS: A Metabolomics Study
Source: Metabolites. 2022 Aug 19;12(8):763. doi: 10.3390/metabo12080763 (PMC9414699; doi:10.3390/metabo12080763)
Supplement: Supplementary file 1 [file metabolites-12-00763-s001.zip › metabolites-1856568-supplementary.pdf]

metabolites

Supplementary data

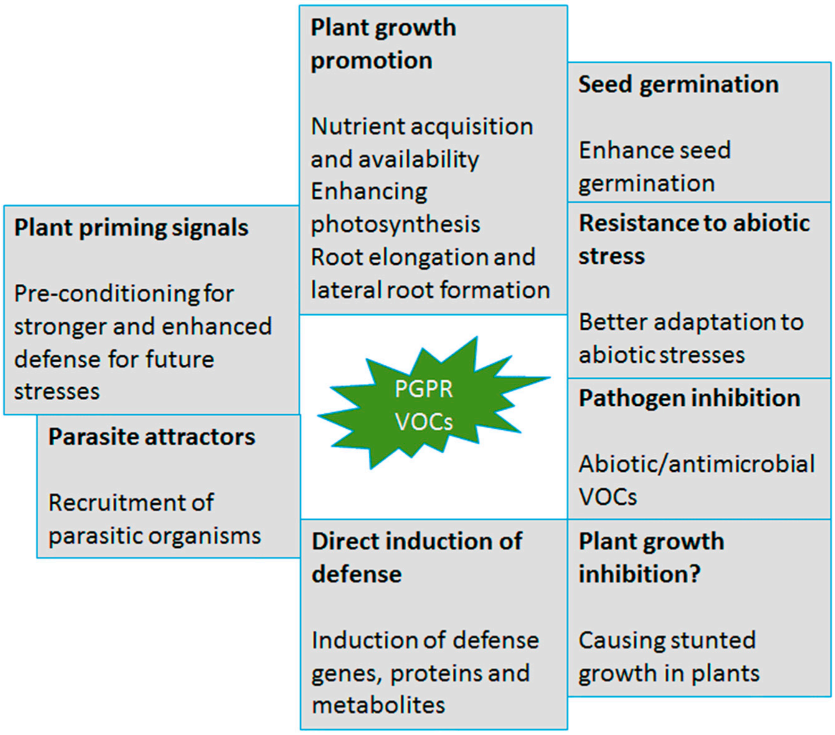

Figure S1. Action mechanisms of PGPR-derived VOCs. PGPR-emitted VOCs can influence different plant physiological processes in various ways. Some VOCs are able to inhibit germination and growth of plant pathogens in vitro or repel herbivores and attract herbivore parasitoids on infested plants. VOCs can impact on plant defensive systems by inducing the synthesis of defense genes, proteins and metabolites (e.g., phytoalexins) that impair microbial colonization. VOCs can also act as priming stimuli by inducing systemic changes and accumulation of transcription factors that may facilitate faster expression of plant defenses, thereby enhancing tolerance or resistance to a future stress.

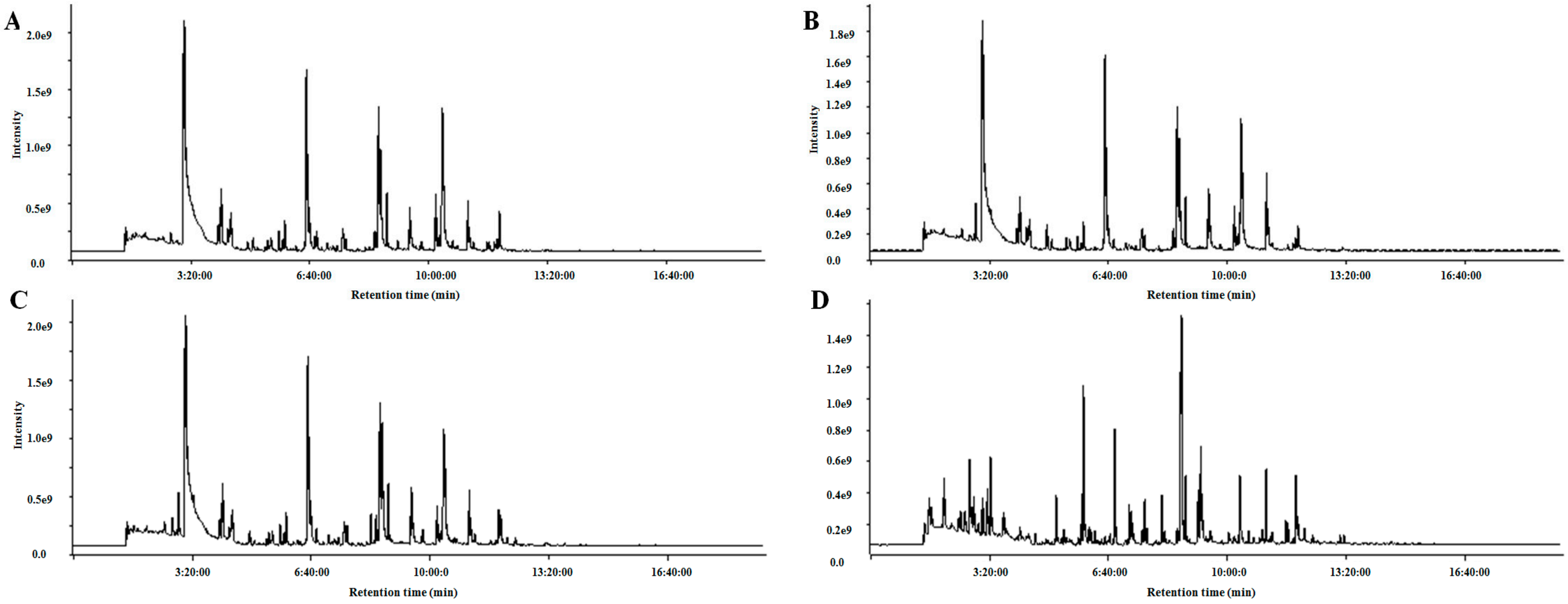

Figure S2. Representative GC-TOF-MS chromatograms of PGPR volatiles sampled by head-space SPME. GC-TOF-MS chromatograms displaying comparative chromatographic differences, and visual inspection thereof evidently shows differential peak populations. (**A**): *Pseudomonas fluorescens* N04, (**B**): *Pseudomonas koreensis* N19, (**C**): *Lysinibacillus sphaericus* T19 and (**D**): *Paenibacillus alvei* T22.

metabolites

MDPI

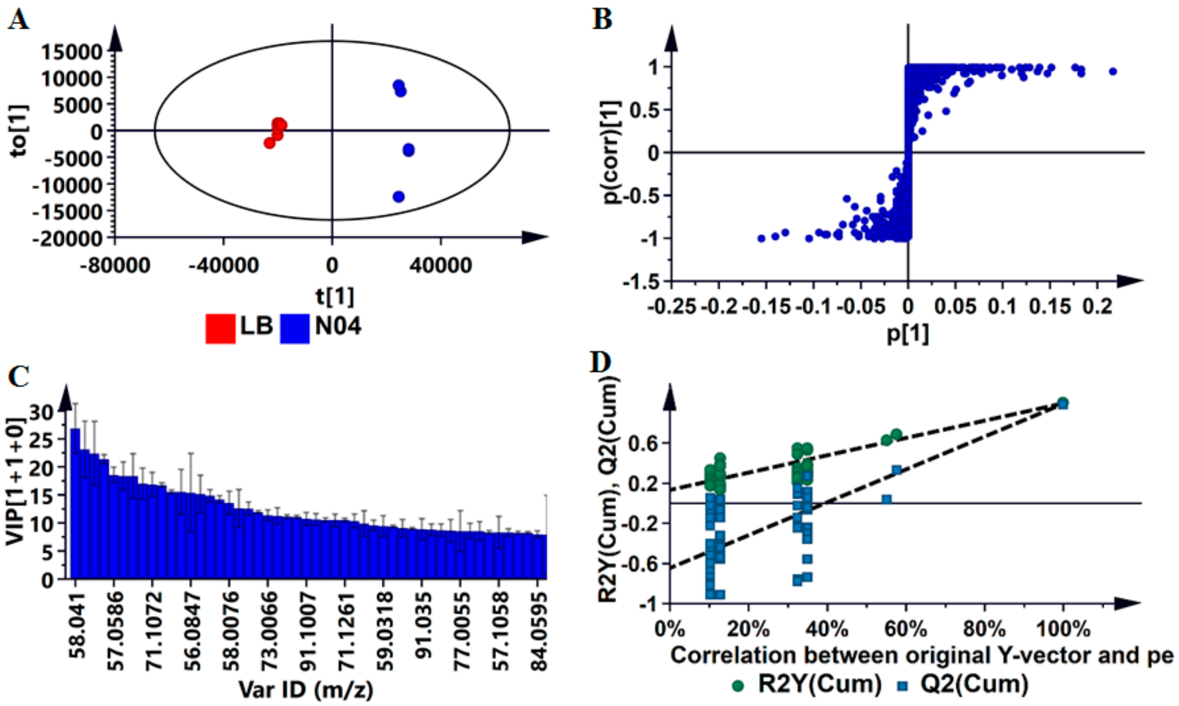

Figure S3. OPLS-DA modeling and variable/feature selection of *Pseudomonas fluorescens* N04 data acquired on GC-TOF-MS. (A): A typical **OPLS-DA score** separating un-inoculated media (LB) *vs.* inoculated media (N04) (1 + 1 + 0 components, $R^2X = 0.895$, $Q^2 = 0.999$, CV-ANOVA p-value = 1.3×10^{-19}). **(B)**: An OPLS-DA **loadings S-plot** for the same model in **(A)**; only variables with the correlation *[p(corr)]* of ≥ |0.5| and covariance of *(p1)* ≥ |0.5| were chosen as discriminating variables and identified using the *m/z* to generate elemental composition. **(C)**: A **variable importance for the projection (VIP)** plot for the same model, pointing mathematically to the importance of each variable in contributing to group separation in the OPLS-DA model. **(D)**: **The response permutation test plot** (n = 50) for the same OPLS-DA model: R^2 (0.0, 0.145) and Q^2 (0.0 − 0.672) values of the permuted models are represented on the left-hand side of the plot, corresponding to y-axis intercepts.

metabolites

MDPI

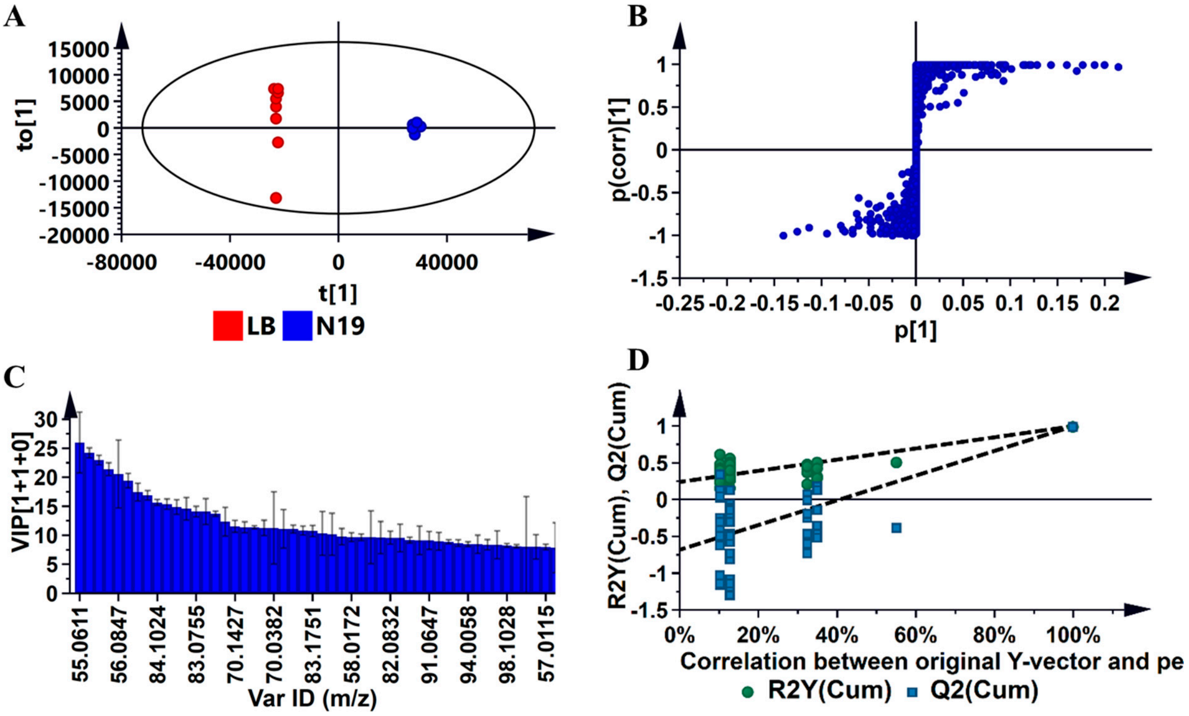

Figure S4. OPLS-DA modeling and variable/feature selection of *Pseudomonas koreensis* N19 data acquired on GC-TOF-MS. (A): A typical **OPLS-DA** score separating un-inoculated media (LB) *vs.* inoculated media (N19) (1 + 2 + 0 components, $R^2X = 0.920$, $Q^2 = 0.998$, CV-ANOVA p-value = 1.1×10^{-12}). **(B)**: An OPLS-DA **loadings S-plot** for the same model in **(A)**; only variables with the correlation *[p(corr)]* of ≥ |0.5| and covariance of *(p1)* ≥ |0.5| were chosen as discriminating variables and identified using the *m/z* to generate elemental composition. **(C)**: A **variable importance for the projection (VIP)** plot for the same model, pointing mathematically to the importance of each variable in contributing to group separation in the OPLS-DA model. **(D)**: The **response permutation test plot** (n = 50) for the same OPLS-DA model: R^2 (0.0, 0.273) and Q^2 (0.0 − 0.606) values of the permuted models are represented on the left-hand side of the plot, corresponding to y-axis intercepts.

metabolites

MDPI

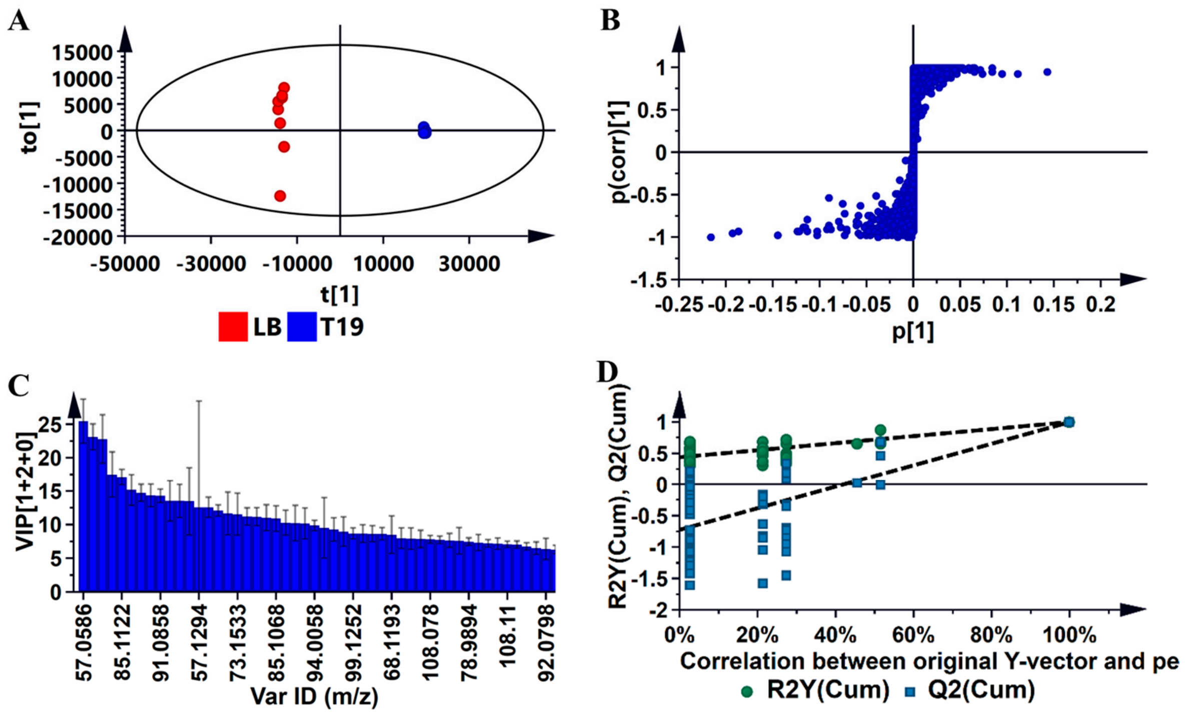

Figure S5. OPLS-DA modeling and variable/feature selection of *Paenibacillus alvei* T19 data acquired on GC-TOF-MS. (A): A typical **OPLS-DA score** separating un-inoculated media (LB) *vs.* inoculated media (T19) (1 + 1 + 0 components, $R^2X = 0.885$, $Q^2 = 0.999$, CV-ANOVA p-value = 1.6×10^{-19}). **(B)**: An OPLS-DA **loadings S-plot** for the same model in **(A)**; only variables with the correlation *[p(corr)]* of ≥ |0.5| and covariance of *(p1)* ≥ |0.5| were chosen as discriminating variables and identified using the *m/z* to generate elemental composition. **(C)**: A **variable importance for the projection (VIP)** plot for the same model, pointing mathematically to the importance of each variable in contributing to group separation in the OPLS-DA model. **(D)**: The response permutation test plot (n = 50) for the same OPLS-DA model: R^2 (0.0, 0.412) and Q^2 (0.0 − 0.757) values of the permuted models are represented on the left-hand side of the plot, corresponding to y-axis intercepts.

metabolites MDPI

Table S1. VOC profiles of PGPR strains: *Pseudomonas fluorescens* (N04), *Pseudomonas koreensis* (N19), *Paenibacillus alvei* (T19) and *Lysinibacillus sphaericus* (T22). Green indicates VOCs primarily present in N04 (but also in N19, T19 and T22); yellow indicates VOCs primarily present in N19 (also in T19 and T22); purple indicates VOCs primarily present in T19 (also in T22); and red indicates VOCs present only in T22.

Rt (min)	Base mass (*m/z*)	Compound name (tentative annotation)	*P.f.*_N04	*P.k.*_N19	*P.a.*_T19	*L.s.*_T22
**Alcohols**						
02:48.3	55.04	1-Pentanol	green	green	green	
03:00.7	45.02	1-Hexanol, 6-amino-			purple	
03:52.7	59.04	1-Hexanol			purple	purple
04:07.0	56.06	2-Decanol	green	green	green	
05:15.6	85.06	Pentanol, 5-amino-				red
08:22.6	57.06	1-Penten-3-ol				red
09:18.3	45.02	2-Undecanol			purple	
09:48.2	43.01	2-Undecanol, acetate	green	green		
10:56.6	45.02	2-Tridecanol	green	green	green	
11:00.4	45.02	2-Tetradecanol				red
11:02.3	55.04	11-Hexadecen-1-ol, (Z)-		yellow		
13:15.9	43.03	1-Hexadecanol				red
16:53.4	71.06	2-Undecen-4-ol				red
**Ketones**						
01:42.1	43.00	2-Propanone, 1-methoxy-			green	green
02:38.8	43.03	2-Butanone, 3-methyl-	green	green	green	
02:59.2	43.01	3-Pentanone				red
04:10.5	43.04	4-Heptanone	green		green	green
06:31.4	43.02	3-Nonanone				red
07:49.3	43.03	6,7-Dodecanedione	green		green	
08:46.7	120.07	Acetophenone, 4'-amino-			purple	
08:54.8	69.04	2-Octen-4-one				red
09:30.7	58.03	2-Dodecanone	green	green	green	green
10:54.3	43.02	2-Nonadecanone		yellow		
11:05.9	43.02	11-Dodecen-2-one	green			
11:47.0	57.06	8-Pentadecanone		yellow	yellow	
12:26.9	58.04	2-Tetradecanone		yellow	yellow	
**Alkanes**						
01:59.3	59.04	Propane, 1-ethoxy-	green			green
01:59.7	57.06	n-Hexane	green		green	
02:31.3	59.04	Butane, 1-ethoxy-	green		green	green
02:53.6	45.01	2-Ethoxypentane			purple	
03:01.4	43.01	Pentane, 1-ethoxy-	green	green		green
03:32.4	59.04	Propane, 1,1-diethoxy-	green		green	green
04:04.8	43.03	Heptane, 2,3-dimethyl-	green			green
04:28.2	43.03	Nonane	green	green	green	green
04:50.9	57.06	Octane, 2,2,6-trimethyl-		yellow		
05:09.6	57.06	Octane, 3,6-dimethyl-	green	green		
06:04.7	117.10	Indane			purple	
07:40.5	43.03	Undecane	green			green
10:09.0	57.06	Tetradecane, 3-methyl-				red
11:11.9	43.03	Hexadecane	green		green	green
11:30.6	91.06	Undecane, 6-phenyl-	green			green
12:45.9	57.06	Heptadecane			purple	purple
13:22.9	57.06	Octadecane	green		green	green
15:17.3	57.06	Heneicosane	green			green
15:33.8	57.06	Nonadecane		yellow		

metabolites

MDPI

15:35.3	43.03	Dodecane, 2-methyl-6-propyl-				
18:07.9	71.09	Triacontane				
18:08.0	57.06	Hexacosane				
**Sulfides**						
02:56.7	94.00	Disulfide, dimethyl				
05:21.0	126.01	Dimethyl trisulfide				
06:34.5	57.07	Sulfone, butyl isopropyl				
07:27.5	91.07	Benzyl methyl sulfide				
**Alkenes**						
04:27.2	91.07	Benzene, 1,3-dimethyl-				
04:44.8	57.06	Benzene, (2-methyloctyl)-				
06:28.8	67.05	1,9-Decadiene				
06:31.7	54.03	1,4-Undecadiene, (E)-				
06:34.3	70.07	1-Undecene				
07:34.5	41.02	Z-1,8-Dodecadiene				
08:29.0	45.02	1-Nonene				
08:58.2	43.03	1-Undecene, 7-methyl-				
09:53.0	71.04	3-Methoxyhex-1-ene				
10:19.8	55.04	7-Hexadecene, (Z)-				
10:33.0	43.03	1,9-Dodecadiene				
11:02.4	55.04	1,13-Tetradecadiene				
**Salicylic acid derivatives**						
07:44.0	120.05	Methyl salicylate				
10:48.3	120.05	Isoamyl salicylate				
11:55.0	120.04	n-Hexyl salicylate				
**Terpenoids**						
05:21.3	93.08	ß-Phellandrene				
05:25.1	93.08	ß-Pinene				
05:46.3	43.98	ß-Ocimene				
05:57.9	68.06	D-Limonene				
06:07.1	91.06	α-Pinene				
06:16.9	93.08	τ-Terpinene				
07:58.0	41.02	Citronellol				
**Aldehydes**						
02:16.5	44.02	Butanal, 3-methyl-				
05:12.3	77.04	Benzaldehyde				
05:37.0	41.02	Octanal				
06:07.0	91.06	Benzeneacetaldehyde				
06:13.7	41.02	2-Octenal, (E)-				
06:42.7	57.05	Nonanal				
07:44.9	43.03	Decanal				
08:08.0	41.02	2,6-Octadienal, 3,7-dimethyl-, (Z)-				
08:35.6	58.11	Hexanal, 2-methyl-				
08:43.8	57.05	Undecanal				
09:38.4	57.05	Dodecanal				
11:19.3	43.03	Tetradecanal				
12:50.2	57.05	Octadecanal				
**Carboxylic acids**						
02:37.8	57.02	Propanoic acid, ethyl ester				
02:43.0	43.04	Butanoic acid, methyl ester				
04:22.0	43.02	Butanethioic acid, S-methyl ester				
08:39.8	127.13	Octanethioic acid, S-hexyl ester				
09:29.5	88.06	Decanoic acid, ethyl ester				
12:36.8	105.05	Benzoic acid, 3-methylphenyl ester				
12:50.2	43.03	Pentadecanoic acid				
13:46.3	43.03	n-Hexadecanoic acid				
14:24.9	88.06	Octadecanoic acid, ethyl ester				

metabolites

**Furans**						
02:07.1	41.02	2(3H)-Furanone, dihydro-3-methyl-				
05:04.7	128.05	2-Methyl-3-(methylthio) furan				
05:30.5	81.04	Furan, 2-pentyl-				
05:37.7	118.06	Benzofuran				
08:05.2	115.05	Furan, 3-phenyl-				
**Amines**						
01:34.9	42.98	2-Methanesulfonylethanamine				
02:01.4	43.97	2-Pentanamine				
02:23.0	43.96	1-Hexanamine				
02:24.7	43.98	2-Octanamine				
02:38.8	61.02	Monoethanolamine				
02:50.2	56.05	2-Propen-1-amine				
03:57.0	41.01	Propylamine				
04:09.6	57.06	1,3-Propanediamine				
04:51.2	41.01	1-Heptanamine				
05:05.0	42.97	1-Butanamine, 3-methyl-				
05:34.8	91.07	Benzeneethanamine				
06:03.2	59.04	Propylamine				
08:34.9	41.70	Ethylenediamine				
10:10.1	73.05	1-Butanamine				
**Pyrazine**						
04:36.8	42.01	Pyrazine, 2,5-dimethyl-				
05:38.5	42.01	Pyrazine, trimethyl-				
06:11.9	121.10	Pyrazine, 2-methyl-5-(1-methylethyl)-				
07:03.8	108.09	2-Isobutyl-3-methylpyrazine				
07:18.0	149.14	Pyrazine, 3,5-diethyl-2-methyl-				
08:16.4	122.11	Pyrazine, 3-butyl-2,5-dimethyl-				
